# Multi-freedom metasurface empowered vectorial holography

**DOI:** 10.1515/nanoph-2021-0662

**Published:** 2022-01-26

**Authors:** Zi-Lan Deng, Zhi-Qiang Wang, Feng-Jun Li, Meng-Xia Hu, Xiangping Li

**Affiliations:** Guangdong Provincial Key Laboratory of Optical Fiber Sensing and Communications, Institute of Photonics Technology, Jinan University, Guangzhou 510632, China

**Keywords:** metasurfaces, polarizations, vectorial holography

## Abstract

Optical holography capable of the complete recording and reconstruction of light’s wavefront, plays significant roles on interferometry, microscopy, imaging, data storage, and three-dimensional displaying. Conventional holography treats light as scalar field with only phase and intensity dimensions, leaving the polarization information entirely neglected. Benefiting from the multiple degrees of freedom (DOFs) for optical field manipulation provided by the metasurface, vectorial holography with further versatile control in both polarization states and spatial distributions, greatly extended the scope of holography. As full vectorial nature of light field has been considered, the information carried out by light has dramatically increased, promising for novel photonic applications with high performance and multifarious functionalities. This review will focus on recent advances on vectorial holography empowered by multiple DOFs metasurfaces. Interleaved multi-atom approach is first introduced to construct vectorial holograms with spatially discrete polarization distributions, followed by the versatile vectorial holograms with continuous polarizations that are designed usually by modified iterative algorithms. We next discuss advances with further spectral response, leading to vivid full-color vectorial holography; and the combination between the far-field vectorial wavefront shaping enabled by vectorial holography and the near-field nano-printing functionalities by further exploiting local polarization and structure color responses of the meta-atom. The development of vectorial holography provides new avenues for compact multi-functional photonic devices, potentially useful in optical encryption, anticounterfeiting, and data storage applications.

## 1 Introduction

Optical holography, the technique for complete wavefront manipulation of light field, has been showing great impacts on various optical applications such as interferometry, microscopy, lensless imaging, data storage, three-dimensional (3D) display and so on, since its invention by Dennis Gabor in 1948 [1]. The word “holography” originally translates as “total recording” from Greek, meaning that it cannot only record the intensity information of light as the usual photography does, but also record the phase information, which plays a crucial role on the reconstruction of a 3D scene of the real world. As electromagnetic field, the amplitude and phase are only partial attributes of light, the vectorial/polarization property is equally important but was always omitted in conventional holography and diffractive optics. To certain extent, the polarization of light has been controlled in conventional holography, namely, polarization holography that can multiplex dual holographic images at orthogonal polarization states by employing bulky photo-induced birefringence materials [2], [3], [4], [5]. However, the potential of full vectorial holography including both versatile polarization states and spatial distributions cannot be entirely exploited due to the limited degrees of freedom (DOFs) in conventional holographic recording medium.

On the other hand, optical metasurfaces with spatially varying nanostructures aiming at versatile wavefront shaping in a compact way have become a current subject of intense research in recent years [6], [7], [8], [9], [10], [11], [12], [13], [14], [15], [16], due to their powerful ability to efficiently modulate the amplitude, phase, and polarization of light at an ultra-thin planar platform. By extensively exploiting the optical responses of meta-atoms with various geometries, sizes and orientations, optical field manipulation based on metasurfaces has evolved from the single-DOF at the beginning to multiple DOFs manipulation nowadays. Based on the single-DOF metasurfaces with pure phase modulation, a variety of planar optical devices have already been developed, including metalens [17], [18], [19], [20], [21], [22], [23], metasurface polarization elements [24], [25], [26], [27], metasurface holography [28], [29], [30], [31], [32], [33], [34], [35], [36] with high performances. As the most general wavefront shaping scheme, metasurface holography has shown superior performances such as high efficiency [30, 31], high fidelity [30, 31], and wide viewing angle [37], [38], [39]. Further based on the multi-DOFs manipulation, multi-functionalities could be integrated in a single metasurface, typically manifesting multiplexing of holographic information based on orthogonal physical channels such as polarization [40], [41], [42], [43], [44], [45], [46], [47], [48], [49], [50], [51], [52], [53], [54], wavelength [55], [56], [57], [58], and orbital angular momentum [59], [60], [61], [62], [63]. Although significantly increased controlling capability has been achieved in multiplexed metasurface holography, the complete reconstruction of a coherent light wavefront with desired arbitrary polarization states and distribution is not realized yet until the proposal of full vectorial holography. Vectorial holography produces holographic images with desired arbitrary polarization states and spatial distributions, in contrast to previous polarization multiplexing holography that has only partially vectorial properties. The first metasurface vectorial holography was demonstrated by interleaving multiple sub-units each of which produces a particular polarization state [64]. Since the interleaved number is limited, the states of encoded polarizations are discretely distributed with a finite total number. Later on, researchers took efforts to achieve spatially continuous polarization distribution upon the holographic image with infinite numbers of encoded polarization states based on various modified iterative algorithms [65], [66], [67]. Further spectral manipulation based on multi-DOF metasurfaces leads to full-color vectorial holography that could display vivid colorful holographic images with well-designed polarization distributions [68, 69]. In addition, the vectorial holography with the capability of far-field manipulation can be readily combined with near-field manipulation capability like grayscale and structure color nanoprint, significantly expanded the application range of metasurface holography. As great progress has been achieved in the field of metasurface holography, many review articles has well summarized about it [70], [71], [72], [73], [74], [75], [76], [77], [78], [79], [80], [81], [82], [83], [84], [85], [86], [87], [88]. In this review article, we will mainly focus on vectorial holography enabled by multi-DOF metasurfaces. The content is organized as follows. In Section 2, we first introduce the vectorial holography based on the interleaving approach; followed by continuous vectorial holography in Section 3. In Section 4, we will discuss the progress on full-color vectorial holography. In Section 5, combined vectorial holography and nanoprint functionality will be included. Finally, in Section 6, we will give an outlook and perspective on the future development of vectorial holography.

## 2 Vectorial holography with discretely distributed polarization states

**Figure 1:**
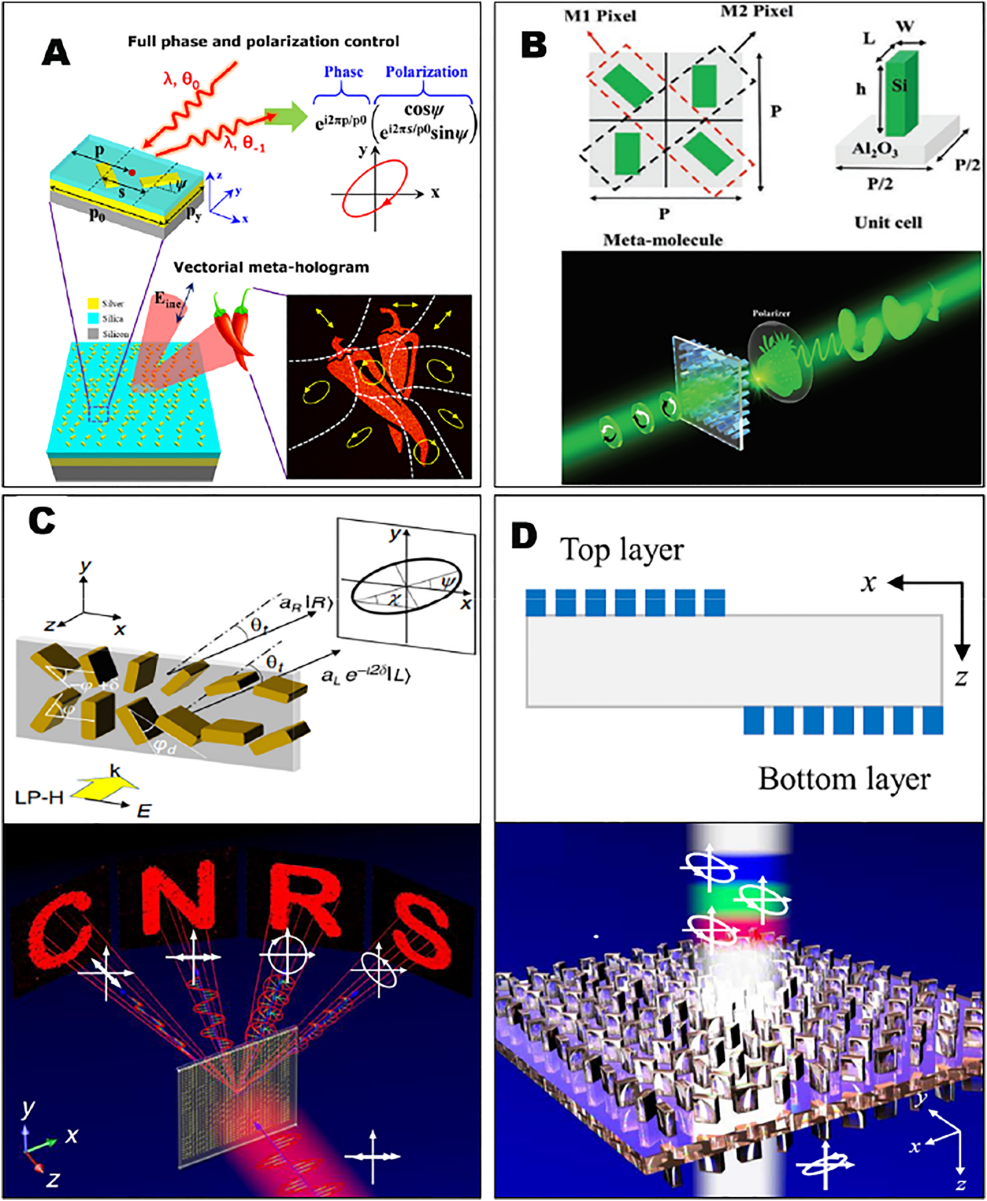
Multi-atomic metasurfaces for vectorial holography. (A) Diatomic metasurfaces for vectorial holography with spatially varying polarizations based on combined geometric phase and detour phase modulations [64]. (B) Tetratomic metasurfaces for switchable vectorial holography under the incidence of RCP and LCP [95]. (C) Pixelated multi-atomic metasurfaces for the full-polarization-reconstructed multi-directional meta-hologram [97]. (D) Angular nondispersive design with pixelated multi-atomic metasurface doublet [98].

To construct the vectorial holography with spatially varying polarization distributions, multi-DOF metasurfaces with simultaneous phase and polarization modulation is required. Deng et al. first demonstrated the vectorial holography by a diatomic plasmonic metasurface. By combining geometric Pancharatnam–Berry (PB) phase and detour phase modulation, it can realize full phase and polarization control of diffracted light independent of the incident wavelength and angle [64]. As shown in Figure 1A, two identical and mutually orthogonal metal nanorods were used to independently control the phase and polarization of impinging light by their relative displacements and orientation angles. It originates from the polarization-selective diffraction mediated by the anisotropic nanorods. Each anisotropic nanorod diffracts light with polarization parallel to its long axis to -1st diffraction order. By arranging two perpendicular nanorods in one unit-cell, orthogonal components of diffracted light can be readily modulated by the parameters of the double nanorods. Based on the detour phase and geometric PB phase modulation rules, the relative phase and amplitude ratio between the two field components are determined by the relative displacement and orientation angle of the double nanorods, while the overall phase is determined by the global displacement with respect to the unit cell boundary. In order to obtain high diffraction efficiency in the -1st diffraction order, a metal–insulator–metal (MIM) metagrating configuration with well controlled gap plasmon response was adopted [38]. Utilizing the powerful ability of simultaneously modulating the phase and polarization states in diffraction by the diatomic unit cell, it is possible to obtain a holographic image with discretely distributed polarization distribution in the -1st diffraction order under the incidence of linear polarized light [64]. One advantage of the diatomic design is that, the meta-atoms mentioned above are all the same in shape and size, with only displacement and orientation modulations, which largely relaxes stringent fabrication requirements. In addition, other approaches employing combined geometric PB phase and propagation phase can also achieve the full phase and polarization control, as realized by a dielectric single-atom metasurface for vectorial holograms. Such dielectric metasurface integrates multiple discrete polarization steering channels for various spatial phase distributions into a single birefringent vectorial hologram, and unwanted crosstalk is effectively suppressed. In this way, multiple independent target phase profiles with quantized phase relationships are realized in a single metasurface, and these phase relationships can handle different information in different polarization states. By selecting the desired combination of input/output polarization states, the reconstructed vectorial holographic image can be alternatively switched with negligible crosstalk [89]. As the employed propagation phase in single-atom dielectric metasurfaces requires multiple sized nanostructures and the phase is highly dependent on the impinging frequency, it suffers from stringent fabrication requirements, narrow bandwidths and limited number of DOFs. The popular way for vectorial holography is the multi-atom approach, which employed frequency-decoupled phase modulations, and the multi-atom design principle can significantly increase the DOF of optical field manipulation, as the variable parameters in a multi-atom unit cell are significantly increased compared with the single-atom unit cell design [90], [91], [92], [93], [94].

As shown in Figure 1B, a tetratomic metasurface was proposed to multiplex a vectorial hologram behind two different holographic images produced by the right circular polarized (RCP) light and left circular polarized (LCP) light. Two independent sets of holograms for the RCP and LCP light are interleaved as the “X” shape in the meta-molecule. An extra vectorial hologram is encrypted in the overlap area of the former two images by encoding the phase difference between the meta-holograms for the RCP and LCP light. When holographic images related to different polarizations overlap, a number of linear polarizations can be realized in the holographic image, and additional information can be decrypted. By continuously changing the phase difference of the incident RCP and LCP, the image in the overlapping area can be modulated [95]. This interleaved tetratomic approach provides additional DOF for multiplexing and encrypting.

To obtain robust multi-DOF optical field manipulation with less fabrication demands and broadband performance, a pixelated metasurface consisting of multiple phase gradient supercells was proposed [96]. As shown in Figure 1C, two rows of phase gradient arrangement of 12 single sized nanobars form a pixelated meta-molecule [97]. The starting orientation angle of both rows results in an effective detour phase that is exploited to address phase information of the meta-holograms, while different orientation angle increment can modulate the phase gradient and polarization of an independent image at a given deflection angle. The dimension of the dielectric building block in the bottom and top lines could be also adjusted to further control the amplitude difference between RCP and LCP light. In this way, the amplitude and phase response of incident RCP and LCP, as well as the diffraction direction could be independently modulated by the supercell pixel. As arbitrary full polarizations covering the Poincaré sphere could be constructed by the dual amplitude and phase modulation based on the circular polarization basis, holographic images with arbitrarily controlled polarization states at designed diffraction directions could be generated under the normal incidence. By interleaving multiple phase gradient supercells with random initial phase, discretely distributed holographic images with linear, circular and elliptical polarizations could be simultaneously produced at different positions of the far field. Based on the phase gradient supercell design with single-sized nanobars, broadband polarization-maintaining vectorial hologram can be constructed [98], as shown in Figure 1D. In such polarization-maintaining metasurface, three rows of phase gradient supercell are employed. To eliminate the frequency-dependent response of different sized nanobars, single-sized nanobars were used and the amplitude of RCP/LCP is modulated by the orientation angle difference between adjacent phase gradient rows, yielding constant polarization parameters (azimuthal angle and ellipticity) over all frequencies. To further eliminate the angular dispersion caused by the high-order diffraction effect, a metasurface doublet formed by two layers of pixelated metasurface can be exploited as shown in Figure 1D. With opposite phase gradient designed along each layer of the metasurface, the angular dispersion can be readily compensated after the incident light passes through both layers. As a result, both the polarization states and propagation direction are maintained for broadband white light illuminations [98]. Generally speaking, increasing the number of meta-atoms in the unit-cell can increase the DOF of light field manipulation. However, the DOF cannot increase endlessly with the meta-atom number, because it is intrinsically limited by other physical constraint, such as the total parameter number existing in a Jones matrix. In addition, increasing meta-atom number may induce other problems such as the design complexity, lower efficiency and strong crosstalk.

## 3 Vectorial holography with continuously distributed polarizations

**Figure 2:**
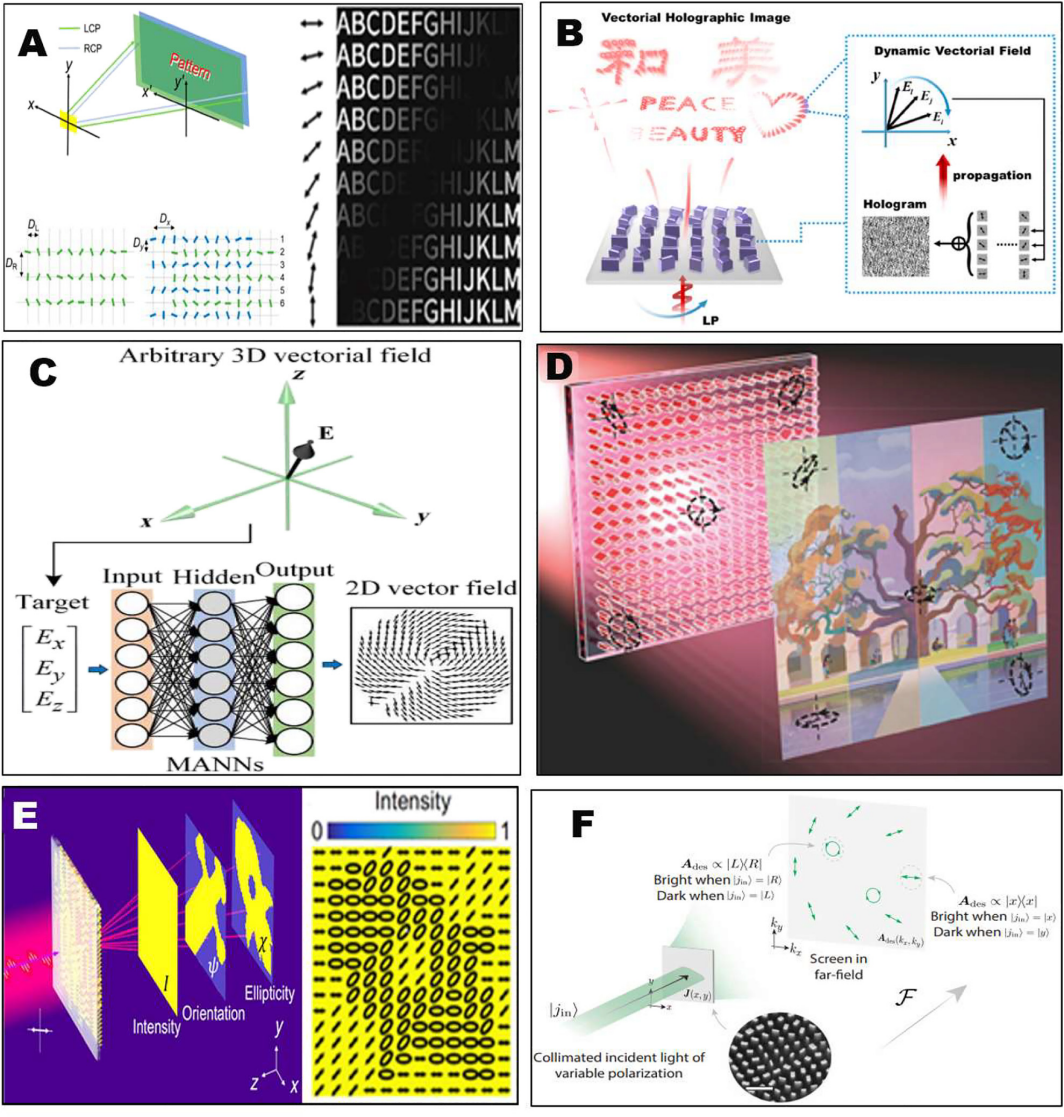
Vectorial holography with continuously modulated polarization distribution. (A) Left: LCP and RCP beams reflected from the metasurface form two identical holographic images but with a spatially continuous phase difference. The unit cell of metasurface. Right: vectorial holographic image with spatial continuous linear polarization distribution [99]. (B) The dynamic display of vectorial meta-holography with 4-fold degeneracy by selecting the desired linear polarization combination continuously [100]. (C) A neural network approach used for constructing a continuous 3D vectorial field in the image space from the 2D vector field distribution in the hologram plane [101]. (D) A metasurface vectorial hologram projecting the full polarization parameters encoding an RGB image [65]. (E) Vectorial Fourier metasurface of arbitrary far-field light distribution of intensity and continuous polarization distribution [66]. (F) The Jones matrix holograms whose far-fields have designer-specified waveplate or polarizer responses [67].

Previous multi-atom interleaving approach for vectorial holography can effectively modulate holographic images with a finite number of polarization states, each polarization states were encoded by a kind of large supercell composed of multiple meta-atoms. In this section, we review continuous vectorial holograms that could modulate an infinite number of polarization states in a more general way. The straight forward way to produce continuous polarization distributions can be realized by superposing two identical holographic images with a spatially continuous phase difference. Figure 2A shows the construction of continuous polarization distribution loaded on a holographic image produced by LCP and RCP incident light. A relative phase difference between the LCP and RCP component is continuously modulated by arranging two sets of nanobars with a relative displacement, while each set of nanobars encodes an identical holographic image by the geometric PB phase. In this way, a continuous varying linear polarization superposed by the two circular polarizations was obtained, which was mixed into the holographic image. Observing the holographic image after a polarizer with different orientation angles, continuously moving shadows upon the image appear, which clearly verified the continuous polarization-varying characteristics of the vectorial holographic image [99] [Right panel of Figure 2A]. In Figure 2B, Zhang et al. combined the resonance effect and geometric phase of the metasurface to simultaneously shape the amplitude, phase, and polarization of light, and build a vectorial hologram with dynamically tuned linear polarization profiles [100]. By optimizing the cross section and the rotation of the nanopillars, five-level amplitude, continuous phase, and polarization modulation were achieved. By sequentially selecting specific input and output polarization combinations, the dynamic display of each vector part on the Fourier plane is realized. To realize the most general 3D vectorial polarization distribution upon the holographic image, Ren et al. resorted to the machine learning inverse design technique [101]. A multilayer perceptron artificial neural network was employed to model the accurate relationship between the arbitrary 3D vector field of the wavefront and the 2D vector field distributions upon the hologram, as shown in Figure 2C. In this way, a vectorial hologram with two digital functions of a phase hologram and a 2D vector field distribution is designed to reconstruct a 3D vectorial holographic image. This 3D vectorial holography allows the lensless reconstruction of a 3D vectorial holographic image with an ultrawide viewing angle of 94° and a high diffraction efficiency of 78%, necessary for floating displays.

Compared with the vectorial holography with continuous-varying linear polarization, the ability to achieve continuous-varying arbitrary polarizations covering the full Poincaré sphere is more challenging. Typically, it requires new hologram algorithms to handle the relationship between the near-field and far-field with respect to not just amplitude and phase parameters, but also the azimuthal angle and ellipticity parameters contained in the polarization information. The whole design procedure acts on the entire metasurface parameters, not just the one by one unit-cell design previously employed by the multi-atom interleaving approach. With this respect, Faraon et al. proposed and employed a modified Gerchberg–Saxton (GS) algorithm to design such vectorial holograms. Because in the polarization of light for fully polarized beams, there are three independent DOFs, in this modified GS algorithm, the amplitudes of both *x* and *y* polarized light, as well as their relative phase are considered. At the beginning of the design procedure, a uniform phase was assigned in the hologram plane. At the following iterative steps, the fields of both polarization components are calculated by the Fourier transforms, and the relative phase between calculated field components should satisfy the predesigned value. To make the iterative symmetric for the two polarizations, the predesigned relative phase is added in the *x* and *y* field components in odd and even steps, respectively. In this way, holographic images with arbitrary polarization states covering the whole Poincaré sphere could be constructed. Based on such powerful capability, they demonstrated the projection of data in arbitrary red-green-blue (RGB) full-color images to the three Stocks parameters (*S*_1_, *S*_2_, *S*_3_) of the vectorial holographic image [65], as shown in Figure 2D.

Afterwards, Song et al. applied such modified GS algorithm under circular polarization bases and realized the continuous-varying vectorial holography based on the pixelated supercell metasurfaces [66]. In this vectorial hologram, arbitrary far-field light distribution with continuous-varying local polarizations, including pre-designed azimuthal angle (orientation) and ellipticity profile is realized, as shown in Figure 2E. Each pixel unit of the metasurface is composed of four rows of phase gradient supercells. The top two rows and the bottom two rows of the supercell are arranged counterclockwise and clockwise, respectively, with the same orientation increment angle. Each building block of the pixel, the pillar element structure, acts as a half-wave plate, converting the chirality of the incident circular polarization, and applying a geometric phase of ±2*δ*, where *δ* is the rotation angle of each pillar (“−” and “+”, respectively, indicate clockwise and counterclockwise rotation). The linear polarization input light can be decomposed into two CP beams, which are deflected to the same angle *θ*_*t*_. The starting orientation angle of the four lines from top to bottom are *δ*_+_, *δ*_+_+∆*δ*_+_, *δ*_−_, and *δ*_−_+∆*δ*_−_, where ∆δ_±_ and δ_±_ are, respectively, used to control the relative amplitude and phase between LCP and RCP. The holographic phase information is, respectively, encoded in LCP and RCP, and arbitrary polarization is realized by the superposition of two CP beams. Because only geometric PB phase are involved in the pixelated metasurface, broadband decoupling of intensity and polarization is readily achieved in such vectorial Fourier metasurface. Therefore, by varying the value of *δ*_±_ and ∆*δ*_±_, arbitrary amplitude and phase information in the metasurface plane can be assigned to each pixel independently from the others, so as to control far-field amplitude and polarization information at will.

Recently, another modified GS algorithm base on Jones matrix was proposed by Rubin et al. Different from previous approaches that only deal with the polarization states of output light, the Jones matrix approach instead seeks to control the polarization transfer function between incident light and output light, and therefore can deal with the most general vectorial holography problem under arbitrary incident light illumination. Therefore, all types of polarization functionalities, such as polarizer and waveplate functions can be merged into holograms, leading to polarizer-type hologram and waveplate hologram. In the iterative process, instead of updating the single-valued scalar quantity, all the four Jones matrix elements were updated based on the Fourier transform. In each step, the Jones matrix ***J*** was decomposed to a Hermitian (lossy, polarizer-like) matrix ***H*** and a unitary (lossless, waveplate-like) matrix ***U*** based on matrix polar decomposition. This decomposition process is the matrix analog of the scalar polar decomposition of a complex number into amplitude and phase. The obtained Hermitian matrix ***H***, and unitary matrix ***U*** play the role of amplitude and phase in the scalar case, respectively. Similar to the amplitude homogenization process in the conventional GS algorithm, the Hermitian part ***H*** of the Jones matrix was discarded, while the overall phase of the Unitary part ***U*** was added to the desired Jones matrix that describe the pre-designed intensity and polarization profiles [67]. For the polarizer-type hologram, each point at the holographic image can be considered as a local polarizer that was able to analyze a pre-designed polarization covering the full Poincare sphere. The local intensity of the holographic image will change according to the analyzing polarization state by the pre-designed spatially varying local polarizers, as shown in Figure 2F. For the waveplate-type hologram, the metasurface is designed to diffract light into a disk in the far-field of uniform intensity. Each point in the disk is designed to implement a different waveplate operation whose retardance varies from 0 to π along the radial direction and fast-axis orientation varies in the azimuthal direction.

## 4 Full-color vectorial holography

**Figure 3:**
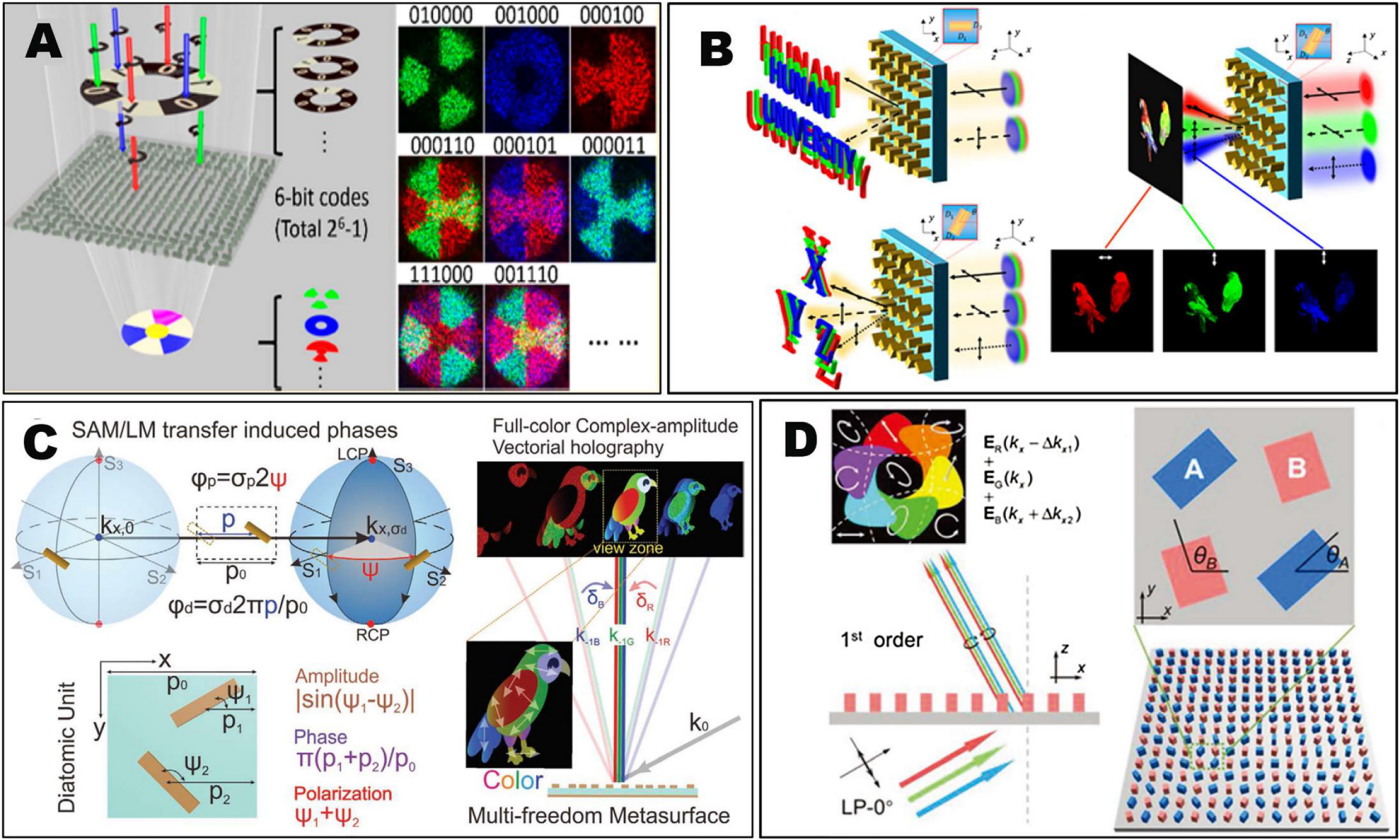
Full-colored metasurface vectorial holography. (A) A multitasked metasurface with noninterleaved single-size Si nanobrick arrays and minimalist spatial freedom demonstrating massive information on simultaneously color and polarization encoded holograms [113]. (B) A noninterleaved metasurface used to realize colorful vectorial holography with trichromatic colors and tripolarization channels [114]. (C) A full-color complex-amplitude hologram that simultaneously controls the amplitude, phase, and polarization, and multiplexes the wavelength [68]. (D) Left: the full-color full-polarization holographic images upon the illumination of linearly polarized laser beams with red, green, and blue (RGB) colors, generated by vectorial and *k*-space engineering. Right: the tetratomic metasurface and a tetratomic macro pixel [69].

In addition to the vectorial holography realized in a single wavelength, multi-wavelength controlled holography is more fascinating as the vivid full-color holographic display can be anticipated. Conventional color holography [102] usually suffers from poor image quality, limited color gamut, and narrow viewing angle [103]. Thanks to the flexible optical field manipulation capability with different spectral responses, metasurface provides a novel solution for producing high-performance color holograms [104], [105], [106], [107], [108], [109]. Color holograms realized on metasurfaces can actually be regarded as information multiplexing at multiple wavelengths. Light with three primary colors (red, green and blue) wavelengths is selected as the basic information carrying channels for wavelength multiplexing. Through resonant tuning of individual meta-atoms [110, 111] or angular dispersion engineering [112], image components for different colors can be redirected into the right positions to form the desired full-color image. To make the colorful holographic image have vectorial properties, the simultaneous control of polarization and wavelength is essential. With this respect, Lin et al. demonstrated a spin-wavelength encoding approach that allows 6-bit control of incident light in both polarization and wavelength dimensions [113]. As shown in Figure 3A, both of the two circular polarization channels and three primary color channels (R, G, B) were exploited to store the holographic information based on a multi-wavelength GS algorithm. As a result, a total (2^6^–1) information storage units could be achieved. Such approach could be readily extended to design versatile wavelength-multiplexed vectorial holographic devices for color display, optical encryption and encoding. As shown in Figure 3B, a tri-polarization channel and trichromatic holography were constructed in a noninterleaved way. Different holographic image combinations are designed for different combinations of input–output polarization pairs. And the three primary color channels are coupled into the three independent polarization pair channels to achieve the full-color vectorial holography, resulting in high-quality and high-efficiency vectorial meta-holography with the large field of view (FOV) in the whole visible regime [114].

Previous colorful vectorial holography only involves a few controlled polarization states for multiplexing. In order to achieve the spatially varying polarization distribution above a full-color image, Deng et al. proposed a multi-DOF metasurface that can simultaneously control the phase, amplitude, polarization and spectral responses of light [68]. A full-color complex-amplitude vectorial hologram has been achieved, which was able to construct spatially varying linear polarization profiles above a full-color holographic image, as shown in Figure 3C. This approach adopted a diatomic unit-cell under the metagrating configuration, the amplitude, phase and polarization of the diffracted light in the -1st diffraction order can be analytically modulated by the orientation angle difference, displacement sum and orientation angle sum, respectively. Different color components of the holographic image were arranged by *k*-space engineering of the angular dispersion.

Subsequently, full-color vectorial holography with spatially-varying full polarization profiles beyond linear polarization was achieved by Guo et al. as shown in Figure 3D. In their approach, full-color holographic images were multiplexed with arbitrary polarization channels through vector superposition and *k*-space engineering based on the geometric phase metasurface of four-atom macros-pixels [69]. All the DOFs in the polarization including its azimuthal angle and ellipticity were completely controlled by the full-color vectorial hologram. Due to the wavelength independent feature of the geometric phase modulation, the three reconstructed color components preserve the same polarization when the selected meta-atom meet the constant phase retardation at the RGB wavelengths. Consequently, the metasurfaces enables the reconstruction of full-color image encoded with arbitrary polarizations including linear, circular and elliptical polarizations. Due to the full free tuning of polarization and color space, application scenarios such as holographic three-dimensional imaging and information encryption could be anticipated. Note that, when vectorial holography is combined with color, information capacity is largely increased, making it harder to reduce the crosstalk between different wavelengths and different polarizations. Therefore, current work only demonstrated the uniform polarization among the entire holographic image or a holographic image block. But in principle, multiple polarization distributions can be encoded into different areas and different colors of the holographic image.

## 5 Vectorial holography integrated with near-field nanoprint

**Figure 4:**
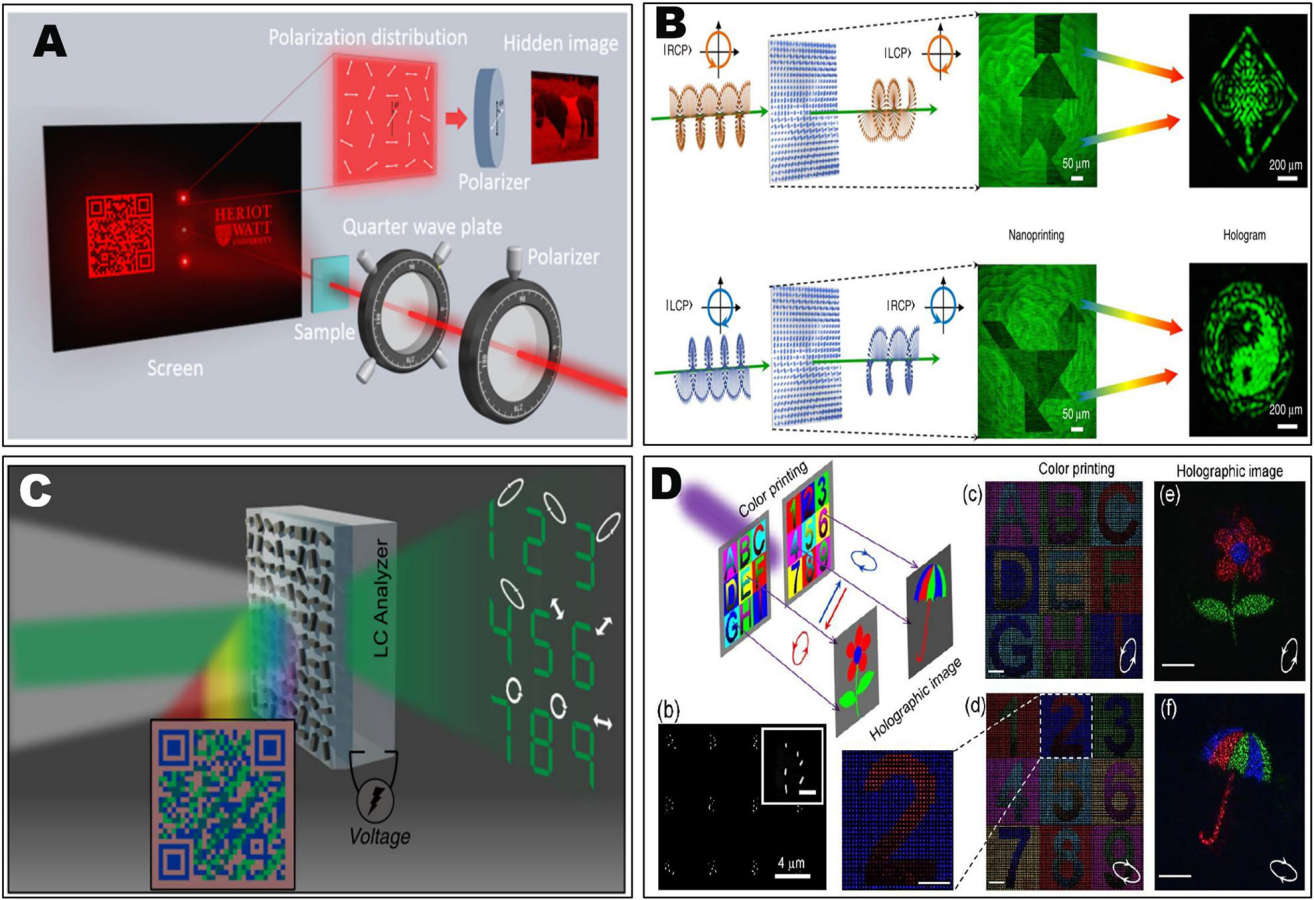
The combination of near-field image encoding and far-field vectorial holography. (A) The multichannel metadevice that combines holographic images and a grayscale image nanoprint for anticounterfeiting and encryption [134]. (B) A four-channel TiO_2_ metasurface device that generates grayscale nanoprinting-hologram images under the illumination of RCP and LCP, respectively, in the near field and far field [135]. (C) A bi-functional metasurface that combines structural color printing and vectorial holography [136]. (D) A silicon metasurface realizes two independent full-color printed images and vectorial holograms [137].

With further increased optical field manipulation DOFs enabled by metasurfaces, vectorial holograph that manipulates the wavefront of far-field light could be integrated with near-field manipulations including grayscale nanoprint [115], [116], [117] and structure color-print [118], [119], [120], [121], [122], open new avenues for information encryption and anticounterfeiting applications [123], [124], [125], [126], [127], [128], [129], [130], [131], [132], [133]. Zhang et al. proposed a multichannel metasurface device that combines the functionality of polarization hologram and spatially varying linear polarization profiles in the near-field based on purely geometric PB phase modulation [134]. This metasurface contains a hologram recording two holographic images for circular polarization components, and at the same time encodes an image in the near-field. As shown in Figure 4A, when illuminating the metasurface with linearly polarized incident light, nonuniform linear polarization profile was generated along the vertical direction for a grayscale image (a horse) encoding. On the other hand, upon the circular polarization incidence, two holographic images [a quick response (QR) code and the University logo] appear in the far-field, which could be seen by naked eyes. The meta-device provides the possibility of high-level anticounterfeiting of compact optical devices with high-density functions.

Exploiting more DOFs in a tetratomic configuration, Liu et al. proposed a multifunctional metasurface that can integrate polarization multiplexed far-field holography and polarization multiplexed near-field nanoprint simultaneously under arbitrary polarization basis [135], as shown in Figure 4B. The metasurface composed of sub-wavelength-spaced TiO_2_ nanopillars. By superposing two types of meta-atoms with simultaneously controlled geometric PB phase and propagation phase, the amplitude/phase under each polarization component was able to be expressed by the orientation angle sum/difference of the two types of meta-atoms, providing a direct guideline to link the meta-atom parameters to required amplitude/phase values of light. In this way, powerful capability of simultaneous and independent control of both phase and amplitude for arbitrary orthogonal polarization state basis can be achieved. Such powerful capability also provides the possibility to point by point modulate the full information of coherent light including its phase, amplitude, polarization azimuthal angle and ellipticity simultaneously and independently with four DOFs. For linear and circular polarization basis, the required meta-atom parameters for a given set of modulated amplitude and phase parameters can be explicitly obtained by analytical formulas. While for the most general elliptical polarization basis, the relationship between the structure parameters and the modulated parameters can be obtained by solving characteristic equations and eigenvectors of the constructed Jones matrix. With such powerful multi-DOF capability, dual-channel near-field manipulation with switchable polarizations, as well as vectorial holographic image displaying in the far field were simultaneously achieved, facilitating compact photonic devices for polarization optics, information encoding, optical data storage, and security.

The combination of vectorial holography with structure color-print was demonstrated by a pixelated metasurface [136]. As shown in Figure 4C, under white light, the device will display a two-colored QR code image in the near field. On the other hand, by coherent light illumination, vectorial holographic images appear in the far-field, and different polarization states can be encoded in different part of the holographic image. This is basically achieved by combining phase modulation and spectral modulation in an asymmetric bar-shaped nano-waveguide. The nanobars act simultaneously as Mie-scatters and truncated waveguide, with the ability to independently modulate phase (waveguide effect) and reflection spectra (Mie-scatterer). Such simultaneous modulation would lead to bi-functional metasurfaces that integrate structural coloring and holographic imaging into a single device. By further integrating the metasurface with the liquid crystal (LC) analyzer, electrically tunable vectorial holographic color printing was realized. Based on this approach, optical information channels have been drastically increased promising for advanced optical encryption platform. Based on the independent control of the RGB intensity and phase, another metasurface that could simultaneously exhibit arbitrary hue, saturation, brightness (HSB) color nanoprinting and a full-color hologram image was proposed [118]. A resolution ∼36,000 dpi can be achieved in their HSB color printing. Their findings open up possibilities for high-resolution and high-fidelity optical security devices as well as advanced cryptographic approaches. Meanwhile, the structure-color integrated vectorial holography was also demonstrated in a silicon metasurface with the capability of independent control of the complex-amplitude in elliptical polarization basis [137]. As shown in Figure 4D, in any orthogonal elliptical polarization state pairs, two independent full-color printing images and vectorial holographic images were simultaneously encoded in a single-layer metasurface. For two holographic images, an RGB flower and umbrella were designed, which can be reconstructed at the predesigned elliptical polarization incidence. While in the near-field area, structure color images demonstrating English letters and Arabic numbers were revealed at the two elliptical polarization states. With such capability, it is potentially useful for future imaging technology, optical data storage, and complex field generation.

## 6 Conclusions and perspective

In conclusion, we have overviewed recent progress on the metasurface empowered vectorial holograms. Vectorial holographic images with spatially varying polarizations were first constructed by multi-atom metasurfaces with flexible phase and polarization control. Then, versatile continuous polarization distributions upon holographic images were realized with the help of modified iterative algorithm including modified GS algorithm, Jones matrix GS algorithm, and machine learning algorithm. Beyond the single-wavelength vectorial holography, full-color vectorial holograms were also constructed by simultaneously modulate the phase, polarization and frequency by meta-atoms. With further intensity or frequency response modulation in the near-field, far-field vectorial holographic image encoding and near-field nonprinting can be combined in the same metasurface layer, largely increased the functionality integration degrees.

Compared with conventional scalar holography, vectorial holography represents the ‘real’ complete recording of light wavefront by including its inherent polarization nature, largely increased the information capacity carried by the light wavefront. The hidden images revealed by the polarization profile demonstrate new information encoding capabilities, providing new avenues on optical encryption and anticounterfeiting applications. With additional frequency DOF provided by metasurface, the full-color vectorial holography was capable of producing vivid colorful stereo images, promising for novel color display elements. The integrated hologram-nonprinting multi-functionalities further increased the information capacity, promising advances on full space vectorial wavefront manipulation.

Based on the emerging vectorial holography technology, developing practical compact optical elements integrating both polarization and diffraction functionalities will be an important research topic in the years ahead. Replacing cascaded optical diffraction elements and optical polarization elements in traditional bulky optical system, optical vectorial diffraction elements constructed by vectorial holography technology will significantly miniaturize the optical system, promising portable optical products in our daily life. In addition, dynamic tunability in metasurface vectorial holography is another important issue and a huge challenge that would be intensively exploited in the upcoming researches. The dynamic modulation will be the most challenges in the vectorial holography field. The functionalities of most current metasurface holograms are static after design. An ideal and universal method for realizing dynamic metasurface holograms is to control the interaction between the waves and each nanostructure of the metasurface at high speed. This requires a metasurface-based spatial light modulator (SLM) with desired refresh rate, modulation efficiency and broadband response performance in the optical frequency range. Outperforming conventional SLM with only scalar phase responses, vectorial SLM with arbitrary phase and polarization control in a reconfigurable way could be a revolutionary advance in optical holography community. Although dynamic control technology based on reprogrammable coding metasurfaces [138], phase change materials [139], [140], [141], and electro-optic modulations [142, 143] has been proposed, it is still very difficult to obtain arbitrary adjustable wavefront modulation in real time and achieve dynamic display of visible light. With the development of nanofabrication technology and the gradual maturity of metasurface design strategies, we believe that those problems will be solved in the near future.
